# The Effect of Bright Light Treatment on Rest–Activity Rhythms in People with Dementia: A 24-Week Cluster Randomized Controlled Trial

**DOI:** 10.3390/clockssleep3030032

**Published:** 2021-09-13

**Authors:** Eirin Kolberg, Ståle Pallesen, Gunnhild Johnsen Hjetland, Inger Hilde Nordhus, Elisabeth Flo-Groeneboom

**Affiliations:** 1 Department of Clinical Psychology, Faculty of Psychology, University of Bergen (UiB), 5009 Bergen, Norway; Gunnhildjohnsen.Hjetland@fhi.no (G.J.H.); inger.nordhus@uib.no (I.H.N.); Elisabeth.Flo@uib.no (E.F.-G.); 2 Department of Psychosocial Science, Faculty of Psychology, University of Bergen (UiB), 5015 Bergen, Norway; Staale.Pallesen@uib.no; 3 Norwegian Competence Center for Sleep Disorders, Haukeland University Hospital, 5021 Bergen, Norway; 4 Optentia, The Vaal Triangle Campus of the North-West University, Vanderbijlpark 1911, South Africa; 5 City Department of Health and Care, 5007 Bergen, Norway; 6 Department of Behavioural Medicine, Faculty of Medicine, University of Oslo (UiO), 0372 Oslo, Norway

**Keywords:** dementia, nursing homes, bright light therapy, rest–activity rhythms, actigraphy, circadian rhythms, clinical trial

## Abstract

Bright light treatment is an effective way to influence circadian rhythms in healthy adults, but previous research with dementia patients has yielded mixed results. The present study presents a primary outcome of the DEM.LIGHT trial, a 24-week randomized controlled trial conducted at nursing homes in Bergen, Norway, investigating the effects of a bright light intervention. The intervention consisted of ceiling-mounted LED panels providing varying illuminance and correlated color temperature throughout the day, with a peak of 1000 lx, 6000 K between 10 a.m. and 3 p.m. Activity was recorded using actigraphs at baseline and after 8, 16, and 24 weeks. Non-parametric indicators and extended cosine models were used to investigate rest–activity rhythms, and outcomes were analyzed with multi-level regression models. Sixty-one patients with severe dementia (median MMSE = 4) were included. After 16 weeks, the acrophase was advanced from baseline in the intervention group compared to the control group (B = −1.02, 95%; CI = −2.00, −0.05). There was no significant difference between the groups on any other rest–activity measures. When comparing parametric and non-parametric indicators of rest–activity rhythms, 25 out of 35 comparisons were significantly correlated. The present results indicate that ambient bright light treatment did not improve rest–activity rhythms for people with dementia.

## 1. Introduction

Dysregulation of circadian rhythms, including the rest–activity rhythm (RAR), is common in people with dementia. The RAR describes a diurnal pattern in activity, typically in terms of cycles of nighttime sleep and daytime activity [1]. In people with dementia, however, the day–night difference in activity is often severely diminished, and the RAR pattern over time is typically characterized by a high degree of irregularity and fragmentation [2]. This coincides with disordered sleep and behaviors such as nocturnal restlessness and daytime inactivity that can impact the care needs and daytime functioning of people with dementia [3]. Furthermore, loss of stability and periodicity in sleep–wake behavior are thought to reflect a deterioration of the endogenous time-keeping mechanisms responsible for circadian rhythmicity [4].

Circadian rhythms (CR) are 24 h oscillations present in physiological processes, including hormone secretion, immune function, body temperature, metabolism, and sleep–wake behavior. CR are coordinated and synchronized by the “master clock” of the body, the suprachiasmatic nucleus (SCN) of the hypothalamus [5], which is entrained by the solar day, allowing various systems to respond in a predictive and coordinated manner to the varying environmental demands and inputs. Although circadian rhythms are frequently studied for their role in sleep, they also play an essential role in synchronizing internal physiology, behavior, and responses to external demands [6,7]. Misalignment of circadian rhythms has been linked to a number of processes that may increase the risk of negative health outcomes, including cardiovascular disease, diabetes, obesity, cancer, and psychiatric conditions including mood disorders and psychosis [6,7].

Multiple lines of evidence indicate a disruption of circadian rhythms in old age and especially in dementia [4,6]. Age-related changes, such as decreased circadian rhythm amplitude (day–night difference), loss of rhythmicity, poor entrainment to the solar day, and internal desynchronization, have been observed for physiological processes, including hormone regulation, core body temperature, and RAR [8].

A disrupted RAR has been linked to behavioral and psychological symptoms of dementia (BPSD) [9], and agitation in dementia patients appears to have a circadian component [10]. Longitudinal studies have found that circadian disruption, including RAR irregularities, may even precede cognitive decline, leading some to hypothesize that disrupted circadian rhythms play a role in accelerating aging and dementia [2,6].

Deterioration of circadian rhythms in old age and dementia has been partially attributed to neural degeneration [11] and partially to lowered exposure to environmental time cues [4]. The most important stimulus to the circadian pacemaker is light, particularly light of short wavelength and high intensity [12,13]. With advancing age, changes to eye physiology impair circadian phototransduction [14]. In addition, lifestyle changes or situational and contextual changes may further reduce daylight availability. For example, people with dementia are often exposed to low levels of environmental illumination, especially those living in nursing homes [15,16,17,18]. Providing a robust environmental time cue through bright light therapy (BLT) is a well-established treatment for disruption of the circadian rhythms in otherwise healthy adults [19,20], but the efficacy of BLT for people with dementia is not yet established. Previous research on BLT in people with dementia has found positive effect on outcomes such as BPSD, cognition, sleep, and circadian rhythms (e.g., [21,22,23,24,25,26]), although studies do not consistently report improvements for all outcomes. BLT is typically administered using light boxes that provide increased illuminance, high correlated color temperature (CCT, i.e., more blue or white in appearance), or both. However, providing BLT using a ceiling-mounted light source eliminates the need to stay with the patient to ensure adherence and allows for longer daily exposures. There is some indication that use of ambient BLT, in particular, may improve circadian rhythmicity for people with dementia [22,27,28], but results have so far been mixed [24,29,30,31]. One reason for diverging results is large variations in methodology, including light parameters (illuminance and CCT or spectral power distribution), exposure time, delivery method, sample size and sample characteristics, trial duration, trial design, and outcome measures [32,33,34].

In the present study, we present a primary outcome from the DEM.LIGHT trial; a 24-week cluster randomized controlled trial to assess the effect of ceiling-mounted BLT for nursing home patients with dementia. Our hypothesis was that the RAR would improve in the group receiving BLT compared to the control group.

## 2. Materials and Methods

The DEM.LIGHT trial (full trial name: “Treatment Light Rooms for Nursing Home Patients with Dementia–Designing Diurnal Conditions for Improved Sleep, Mood, and Behavioral Problems”, ClinicalTrials.gov Identifier: NCT03357328) is a cluster randomized placebo-controlled trial that was conducted in Bergen, Norway, between September 2017 and April 2018. Data was collected at baseline and at 8, 16, and 24 weeks.

### 2.1. Participants

Eligible nursing homes were identified with the assistance of Bergen municipality. Any nursing home was eligible if it had a dedicated dementia unit, the architecture allowed for installation of light panels, and the unit was not currently participating in other projects. Fourteen nursing home dementia unit leaders were invited, of which eight agreed to allow their unit participate in the trial. Four units declined to participate, and one unit signaled interest after the desired number of units was achieved. One unit was excluded due to having twice as many residents as other units. A total of 78 residents lived in the included units and were screened for inclusion (see Table 1 for eligibility criteria) by clinical psychologists (EK and GJH) in collaboration with the nursing home physician.

### 2.2. Sample Size and Power Calculation

The power analysis indicated that a minimum of 64 participants and 8 clusters were needed in order to detect differences between conditions [35,36] using ANOVA analysis. Alpha level was set to 0.05 (two-tailed), and the power, to 0.80, expecting moderate effect sizes (Cohen’s d = 0.50). Allowing for a 20% dropout, the aim was to recruit 80 participants.

### 2.3. Delivery of the Intervention

**Figure 1:**
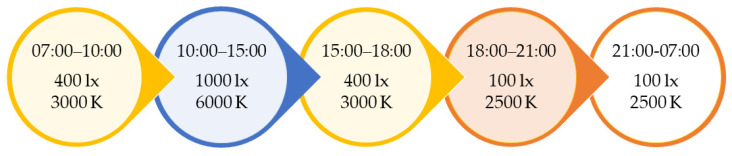
Illuminance (lx) and correlated color temperature (kelvin, K) at different times of the day in the intervention group, with gradual transition periods of 30 min separating each phase. Between 21:00 and 07:00 the lights could also be turned off by staff if this was preferred.

Four units (intervention group) had ceiling-mounted LED panels (Glamox, 1 × C95 48 CCT 6500 MP 47 W/4702 lm, Glamox, Keila, Estonia) installed in the living rooms. The panels were programmed to mimic daily variations in the natural light cycle, delivering light at varying illuminances and CCT throughout the day (see Figure 1). Peak illuminance (1000 lx at eye level) was delivered between 10 a.m. and 3 p.m. each day. The CCT during this period was set to be around 6000, which is within the interquartile range for natural daylight across various atmospheric conditions, i.e., 5712–7757 K [37]. In the control group (four units), lights bulbs were changed (CFL AURA UNIQUE-D/E LL 18W/830 G241-2 in three units and CFL AURA UNIQUE-L LL 18W/830 2G11 in one) but still delivered standard indoor illumination (~150–300 lx, 3000 at eye level in the center of the room).

### 2.4. Group Allocation and Blinding

The eight nursing home units were randomized to the intervention (four units) or control condition (four units) by EK and EF using random group assignment in IBM SPSS Statistics. All participants in a unit were assigned to the same condition. Light bulbs were changed in the control units by researchers in order to conceal condition assignment and to ensure similar light across control group units. Employees at the nursing homes were informed that the aim was to investigate the effect of different kinds of light but were not told which aspects of the light we would be studying. Blinding of residents was not considered an issue due to the degree of cognitive decline experienced by those in the target population.

### 2.5. Measurements

#### 2.5.1. Rest–Activity Rhythms

Movement patterns were assessed using wrist-worn accelerometry devices (Actiwatch II, Philips Respironics Inc., Murrysville, PA, USA) known as actigraphs, which allow for continuous recordings of motor activity under naturalistic conditions. Actigraphy has been validated for the detection of RAR, including for people with dementia [1]. The actigraphs were worn continuously for seven days at each data collection point. As recommended by Camargos et al. [38], actigraphs were placed on the wrist of the dominant arm, epoch length was set to one minute, and the wakefulness threshold was set to medium. Actigraphy data were exported from Actiware (version 6.0.9, Philips Respironics Inc., Murrysville, PA, USA).

Previous research has utilized a variety of methods for characterizing RAR, with non-parametric approaches and cosine-based models being popular [2,39]. As the standard cosine model often shows poor fit with the true shape of the RAR [39,40,41], RAR indicators were computed using a non-parametric approach [42], as well as an extended cosine model [41].

Non-parametric indicators are used to describe the RAR patterns without making assumptions about the shape of the rhythm [42,43]. They have previously been used to describe RAR disturbances in dementia patients [9,43,44,45,46,47], and to evaluate the effect of BLT in such patients [22,24], with good sensitivity [40]. Non-parametric RAR indicators (inter-daily stability, intra-daily variability, least active 5 h, most active 10 h, and the relative amplitude) were calculated from raw actigraphy data using the NparACT package in R [48].

***Inter-daily stability (IS)*** quantifies the consistency of the activity profile from day to day, defined as the ratio of the variability within a 24 h period to the total variability.

$\mathrm{IS}=\frac{n\sum_{h=1}^{p}\;{\left({\bar{X}}_{h}-\bar{X}\right)}^{2}}{p\sum_{i=1}^{n}\;{\left(X_{i}-\bar{X}\right)}^{2}}$ with *n* = the total number of data, *p* = the number of data per day, $\bar{X}$ = the overall mean of all data, ${\bar{X}}_{h}$_=_ the hourly means, and *X_i_* = the individual data points. The resulting value has a range of 0 (Gaussian noise, no similarity between days) to 1 (perfect stability and similarity between days). A high IS, therefore, indicates that different levels of activity occur at similar times across days.

***Intra-daily variability (IV)*** measures the degree of fragmentation within 24 h periods, defined as the ratio of hour-to-hour variability to the overall variability.

$\mathrm{IV}=\frac{n\sum_{i=2}^{n}\;{\left(X_{i}-X_{i-1}\right)}^{2}}{\left(n-1\right)\sum_{i=1}^{n}\;{\left(X_{i}-\bar{X}\right)}^{2}}$. A perfect sine wave has an IV of near zero, while Gaussian noise has a value of about 2 (or sometimes higher). A larger IV indicates a higher number or magnitude of transitions between activity and inactivity, typically reflecting frequent daytime naps or nighttime awakenings.

***L5 and M10*** reflect the average activity levels during the least active consecutive 5 h and the most active consecutive 10 h, typically occurring during the night and day, respectively.

***Relative amplitude (RA)*** is the ratio of the difference in activity level during the most active 10 h (M10) and the least active 5-h period (L5) to the total activity in these two periods.

$\mathrm{RA}=\frac{\left(M10-L5\right)}{\left(M10+L5\right)}.$ A high RA thus indicates a robust rhythm with relatively higher activity in the active period and rest during the inactive period.

In addition, we estimated RAR using the anti-logistic extended cosine model [10,41], which builds on the traditional cosine models. The additional parameters allow for a more flexible fit to activity rhythms that do not conform to sinusoidal patterns [10]. This approach has, therefore, been utilized in research with older people and dementia patients (e.g., [49,50,51]). Extended cosine parameters were calculated using the RAR package [52] in R.

***Amplitude*** represents the difference in activity between the peak (maximum) and nadir (minimum), in other words the magnitude of the rhythm.

***Midline-estimating statistic of rhythm (MESOR)*** captures the mean level of activity (i.e., minimum + amplitude/2), with higher levels indicating more overall activity.

***The pseudo-F statistic*** measures the goodness of fit to the periodic function. Higher values indicate more regular activity patterns that can be modeled by a function with a 24 h period, indicating a robust daily activity rhythm.

***Acrophase*** is the time of peak activity level. Later or earlier times may reflect a delayed or advanced circadian phase, respectively.

***Alpha*** describes the relative width of the trough and peak of the rhythm. Larger values indicate relatively more activity during the rest period than the active period, resulting in wider troughs and narrower peaks.

***Beta*** represents the steepness of the rise and fall of the curve. Larger values indicate steeper curves, i.e., sharper transitions between rest and activity, resulting in a curve with a “squarer” shape.

***Nadir*** is the time at which the activity curve reaches its minimum value.

#### 2.5.2. Light Measurements

The number of light panels needed to provide the required intervention illuminance was calculated by Glamox engineers for each unit before installation. In addition, light was measured after the start of the trial by researchers using the GL Spectis 1.0 T Flicker spectrometer (GL Optic, Puszczykowo, Poland). As recommended by Spitschan et al. [53], the spectral distribution of the light was measured from an observer point of view (vertically at 1.2 m height, to approximate corneal illuminance for a seated patient), and α-opic irradiances were calculated with the CIE S 026 toolbox [54], in addition to photopic illuminance (lx). Data on melanopic equivalent daylight (D65) illuminances (EDI) were also extracted from the toolbox. All measurements were conducted on an overcast day in September between 10 a.m. and 3 p.m., in four directions (facing the wall with the most windows, and at 90-degree steps relative to it).

#### 2.5.3. Other Measurements

To approximate light exposure, staff estimated how much time, on average, the patient had spent in the living room each day between 10 a.m. and 3 p.m. (i.e., the period of peak illuminance and CCT in the intervention condition) since the last data collection point. Patients’ medical journals were accessed by authors with clinical authorization in order to extract information about diagnoses and medications. The Charlson Comorbidity Index (CCI) assesses the number of comorbid conditions, weighted by the seriousness of the disease, and its scores are positively associated with 1-year mortality rates [55]. The Mini-Mental State Examination (MMSE) is a test of cognitive functions [56], with good reliability and validity in the assessment of cognitive impairment and change over time in patients with dementia [56,57]. It is scored on a scale ranging from 0 to 30, with higher scores indicating better cognitive function. The Functional Assessment Staging Test (FAST) [58] describes the progression of Alzheimer’s disease in seven stages from 1 (normal adult) to 7 (severe Alzheimer’s disease). It has demonstrated adequate validity and reliability [59]. Higher stages indicate reduced ability to perform activities of daily living. Such deterioration is not necessarily seen in all forms of dementia, so we only used the scale as a means of characterizing the degree of impairment at baseline.

### 2.6. Data Management and Statistical Analyses

Data analyses were performed in R. Multi-level regression models were fitted using the lme4 library [60] with restricted maximum likelihood estimation, random intercept for each patient, and an unstructured variance–covariance matrix. Amplitude, MESOR, pseudo-f statistic, and beta were transformed using a natural log function (ln) to improve normality of the residual distribution. For the beta model, one extreme outlier had to be removed for the model to be estimated. Significance levels are reported with and without Benjamini–Hochberg false discovery rate correction [61] in order to account for increased risk of a type 1 error when performing multiple tests. Associations between RAR outcomes at baseline were investigated using Spearman correlations.

Scores on the functional assessment staging test (FAST) at baseline were added to control for dementia severity, following the recommendations of Forbes et al. [34]. The following covariates were also tested: age, composite score on the CCI, gender, number of psychotropic medications, and whether the patient was on hypnotic/sedative medications. As they did not change the interpretation of the outcomes, they were not included in the final model.

Patients in either group who had spent less than 30 min on average per day in the living room during the main period of the intervention (10 a.m. to 3.p.m.) were excluded from the analyses.

Acrophase is reported in terms of decimal hours (minutes expressed as the percentage of a full hour). As the effect of light on the timing of RAR might differ for phase advanced and phase-delayed patients, the sample was split according to their deviance from the average acrophase of healthy adults (i.e., 12:59) as reported in a previous study [62]. However, only two patients had a negative deviance from the reference point of 12.59, and sub-group analysis was, therefore, not performed. Rather, these observations, as well as the subsequent observation from the same patient, were removed, amounting to a total of 4 observations (from 2 patients) removed.

### 2.7. Ethical Considerations

Informed consent was provided by legal guardians on behalf of the patients. Patients who were potentially able to understand, as identified by the nursing home physician, were informed in a personally adapted manner and given the option to consent or decline to participate. Any verbal and non-verbal expressions of distress or unwillingness to participate in data collection procedures expressed by the patients were regarded as withdrawal of consent. Patients could freely withdraw to other areas if they were uncomfortable with the light installed in the living rooms.

## 3. Results

### 3.1. Sample Descriptive Statistics

**Figure 2:**
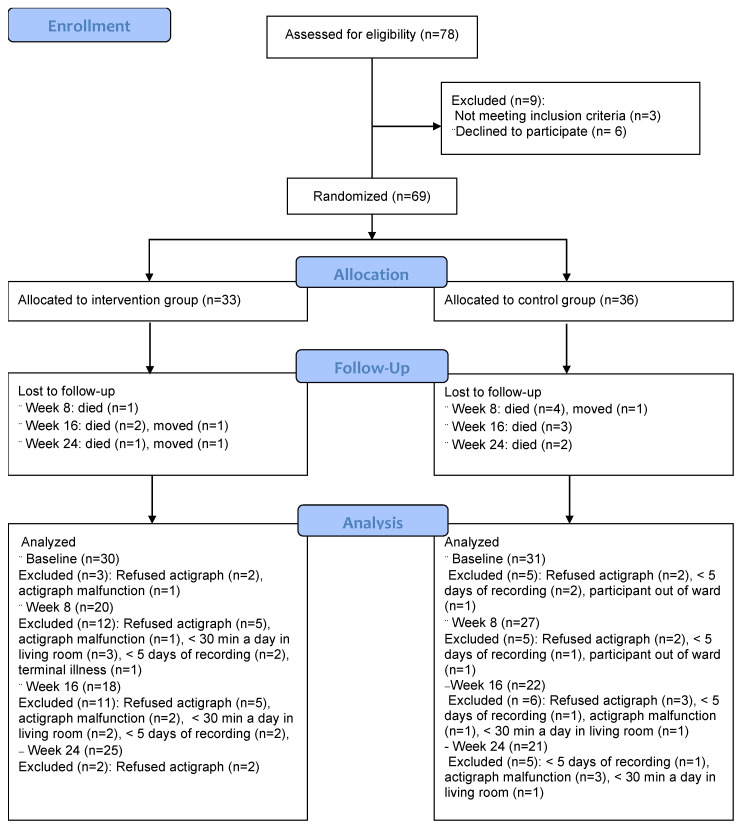
Flow diagram showing participant inclusion, allocation, and attrition through each stage of the trial until analysis.

Eight dementia units at separate nursing homes, with 78 residents in total, were included at baseline. See Figure 2 for a diagram of the participant flow. Three patients were excluded because they did not meet the inclusion criteria (listed in Table 1), and six declined to participate. After allocation, eight more patients were excluded due to absent baseline measurements (details in Figure 2), amounting to 61 with complete actigraphy recordings available for analysis at baseline.

At baseline, 71% of the included patients were women, and the median age was 84 (Table 2). A total of 75% had an MMSE score below 10, indicating severe cognitive impairment [57], and 75% had a FAST score of 6, indicating functional impairment corresponding to moderate Alzheimer’s dementia [58]. All but three patients had a formal dementia diagnosis. Undiagnosed patients were included, as they all had MMSE scores below 26 following assessments by clinicians. In all, 54% of the patients had an Alzheimer’s diagnosis, while the second largest group (31%) was “unknown dementia”. Two patients were diagnosed with Parkinson’s disease.

### 3.2. Adherence

Estimated time spent in the living room during the main intervention period (10 a.m. to 3 p.m.) was on average 3.1 h (SD = 1.4) in the control group and 3.6 h (SD = 1.6) in the intervention group.

### 3.3. Light Measurements

**Figure 3:**
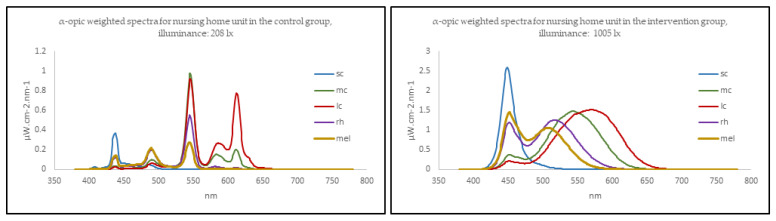
Typical α-opic weighted spectra for nursing home units in the control group (**left**) and the intervention group (**right**). Measured at 1.2 m height, in the center of the room, facing the back wall. Created using the CIE S 026 toolbox [54].

Supplementary Table S1 shows mean illuminance, α-opic irradiance, and melanopic EDI for the two conditions. Figure 3 shows typical examples of α-opic weighted spectra for nursing home units in the control group and intervention group. Spectral distributions are available in supplementary Table S2. Mean illuminance in the intervention group units was 1039 lx in terms of photopic illuminance (SD = 225, range = 722–1242). In the control condition, it was 242 lx (SD = 101, range = 134–368). One nursing home had an average illuminance measurement (722 lx) below the goal of 1000 lx. However, the highest illuminance in the control condition was 368 lx; thus, even the lowest value achieved in the intervention condition was almost double the highest value in the control group.

### 3.4. Rest–Activity Rhythms

Scores on all outcome measures by week of study and treatment group are shown in Supplementary Table S3.

#### Correlations between Circadian Rhythm Outcomes at Baseline

Spearman correlations between non-parametric indicators and indicators from the extended cosine model at baseline are shown in Table 3. A total of 25 of the 35 correlations were significant. There were two very strong (i.e., absolute value above 0.80) correlations: between the pseudo-F statistic and RA (r_s_ = 0.82) and between the pseudo-F statistic and IS (r_s_ = 0.81). Five correlations were strong (i.e., absolute value 0.80–0.79): amplitude and IS (r_s_ = 0.66), amplitude and RA (r_s_ = 0.60), MESOR and M10 (r_s_ = 0.67), nadir and RA (r_s_ = 0.62), and nadir and L5 (r_s_ = 0.66).

**Figure 4:**
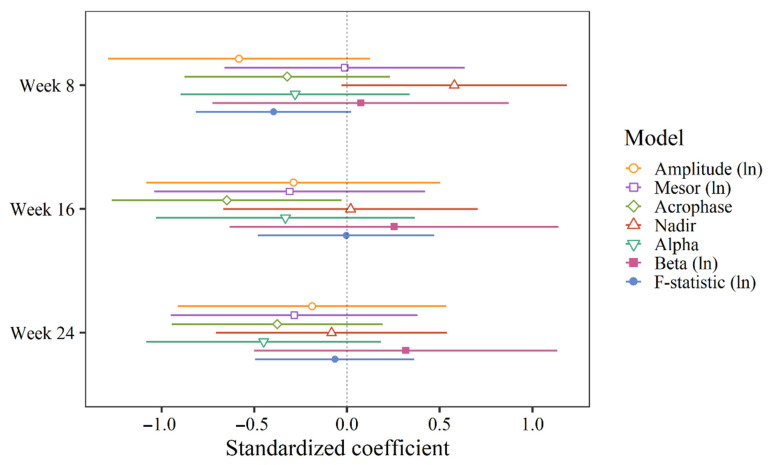
Standardized coefficients for week-by-condition interactions (i.e., change from baseline in the intervention group relative to the control group) for the extended cosine model.

**Figure 5:**
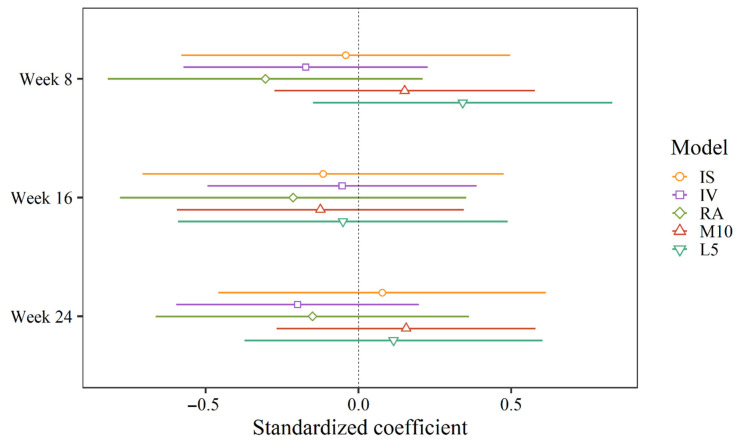
Standardized coefficients for week-by-condition interactions (i.e., change from baseline in the intervention group relative to the control group) for non-parametric indicators.

Table 4 provides an overview of the estimated treatment effects (week-by-condition interactions) for all outcomes. Standardized coefficients, displaying all interaction coefficients on a standardized scale, are shown in Figure 4 and Figure 5. There was a significant difference between the groups in acrophase shift from baseline to week 16 (B = −1.02, 95% CI = −2.00, −0.05). In other words, the mean of the control group was delayed by about one hour from baseline to week 16 compared to the intervention group. In weeks 8 and 24, the control group was delayed by 0.51 and 0.59 h, respectively (i.e., about 30 min) from baseline compared to the intervention group, but this was not sufficient to reach statistical significance (Table 4). With correction for repeated measurements (Benjamini–Hochberg correction), none of the interactions reached statistical significance. There was no significant week-by-condition interaction on any other RAR measure or at any other time point.

## 4. Discussion

The present results do not support the hypothesis of the study, namely that RAR would improve in the intervention group relative to the control group. However, the results do suggest that ceiling-mounted dynamic bright light treatment at nursing home dementia units influenced timing of the activity acrophase after 16 weeks. The group-by-time interaction was, however, not significant for any other time points, RAR outcomes, or after correction for multiple testing.

Although a slight advance of the acrophase in week 16 was observed in the intervention group, the delay in the control group makes up most of the group difference in change from baseline. At the 8- and 24-week follow-ups, the control group was 30 min delayed from baseline compared to the intervention group, but this difference did not reach statistical significance. As week 16 coincided with the months of January/February, it may be hypothesized that the intervention helped prevent phase delay during the darkest months of the year but made less of a difference in April (spring) when more natural light may be more available. An absence of significant group differences at week 8 follow-up in November, which also took place during winter, could indicate that the effect of BLT takes a while to emerge and thus to be detected by significance testing [63].

The efficacy of light in terms of entraining human circadian rhythms, including RAR, has been well documented [19,64], but clinical research with dementia patients has been inconclusive. In line with the present findings, advancement or stabilization of activity acrophase together with non-significant results on other circadian rhythm outcomes have been reported after BLT [31,65,66]. However, a delay of activity acrophase following morning BLT has sometimes been reported [50]. The magnitude and direction of phase shift in response to light depends on the endogenous circadian phase of the recipient [67]. One explanation for the diverging results across studies might, therefore, be variations in the timing of light administration relative to individual circadian time. Studies on BLT in dementia typically do not assess CR by core body temperature or melatonin sampling at baseline, and self-reports or observation are rarely feasible, making it difficult to adequately time the BLT. While an advance of the circadian rhythm with increasing age has frequently been reported [8], studies have suggested a possible association between a delayed acrophase and dementia [51,62,68]. The mean acrophase in the current sample was at 3.35 p.m. (SD = 1.66). Previous studies have found the mean acrophase of older adults without dementia to be around 1 p.m. [62,68], implying that the acrophase in the present sample was somewhat delayed. The sleep schedules of patients were not altered in order to deliver BLT; thus, patients would likely receive the intervention after their natural wake times. Unfortunately, there were not an adequate number of patients with an advanced acrophase to estimate their response to the treatment.

The clinical relevance of affecting acrophase timing is not yet clear. The endogenous circadian rhythm cannot be directly inferred from the rest–activity cycle, as RARs are also subject to environmental influences. Additionally, the relationship between various CR may be altered in old age and dementia [69,70,71], and the endogenous CR might not overlap with the RAR. However, both advanced and delayed acrophase have been linked to mortality [62,72,73], depressive symptoms [74], and cognitive decline [75]. For some individuals, stabilizing or shifting the RAR so that sleep and wakefulness occur at more conventional times might increase opportunities for social participation, ease care-giving, and allow sleep to take place at night when it is less likely to be interrupted. Although also a measure of RAR timing, the nadir did not differ significantly between groups. It may be that the nadir varies less than acrophase due to consistent bedtimes at nursing homes and few opportunities for movement during the night, especially for patients with limited mobility.

Addressing RAR dysfunction in dementia also involves aspects of the RAR beyond acrophase timing, including dampened amplitude, high fragmentation, and the absence of a 24 h rhythm [2]. The present results deviate from a number of previous studies on BLT in dementia that reported improvements on circadian outcomes such as IS [22], IV [22,76], RA [76], the F-statistic [26,50], phasor magnitude (i.e., entrainment to the light-dark cycle, [27,28]), and increased MESOR [50]. However, these studies also report null findings for a number of additional rhythm indicators measured in the same trials. There are also researchers who report no effect of light on any circadian outcome [24,29,30]. In addition to variability in the intensity, composition, timing, delivery method, and duration of the BLT, differences in sample characteristics may partly explain conflicting findings. For instance, old age and advanced neuropathology may entail attenuated responsivity to the effects of BLT [8,14,77]. In the present study, we tested a variety of covariates, including age, comorbidities, gender, and medications, as well as exclusion based on eye disease or use of sedative medications, but did not have sufficient power to perform sub-group analyses. Performing post hoc analyses on patients with circadian disruption at baseline only, or with specific dementia sub-types, was also precluded due to insufficient group sizes. The moderate sample size, along with the large number of possible confounders, represent notable weaknesses of the current study. Although the heterogeneity of our sample entails possible confounding influences, it represents a realistic reflection of a typical nursing home population.

The ultimate goal of providing non-pharmacological treatments at nursing homes is to improve the well-being of the residents. Utilizing outcome measures that accurately reflect the challenges and improvements that are most relevant to the well-being of the residents is, therefore, crucial. We have previously reported that traditional sleep parameters, as measured by actigraphs, were not improved by the current trial [78]. However, previous publications based on data from the current trial found a positive effect of BLT in terms of proxy-rated sleep [78] and depressive symptoms [79]. While reporting biases may play a role regarding the discrepancies between objective and subjective findings, it cannot be ruled out that actigraphy is not optimal for detecting treatment response in individuals who are largely sedentary and whose daily activity rhythms are influenced by nursing home routines.

In the present study, we investigated numerous common indicators of RAR, but the optimal way to capture RAR is a topic of continuing research [2,39]. The two approaches used in the present study yielded correlated, but not equivalent, estimates of RAR (Table 3). Future research is needed to determine which RAR measure has the highest clinical relevance and sensitivity to change. Although actigraphy is commonly used due to the convenience of unobtrusive multi-day measurements, measures based on melatonin and cortisol sampling or core body temperature rhythms may provide a better insight into circadian function. Furthermore, synchrony between various circadian rhythms may constitute an important aspect of circadian function in dementia [69,80]. The use of actigraphy alone is a limitation of the current study, and future studies using multiple measures of circadian rhythms may elucidate the complex interplay between them.

Future research should determine if BLT in combination with other interventions such as melatonin, daytime activity, and nighttime light restriction is more effective than BLT alone [21,81,82]. The current sample was characterized by severe dementia and a high number of comorbidities and medications. While this is representative of the nursing home dementia population, these factors may also interact with or mask the effect of BLT. As circadian disturbance is evident before the onset of cognitive impairment [51,83], targeting at-risk individuals at an earlier stage may also yield different results and should thus be prioritized in future research.

## 5. Conclusions

The results suggest that there was no significant improvement of RAR after 8, 16, or 24 weeks of dynamic ceiling-mounted BLT in nursing home dementia units. However, the control group experienced a significantly larger delay of the acrophase in week 16. More research with larger sample sizes and with subjects with less severe dementia is needed in order to establish the efficacy of BLT on CR disruption in dementia patients.

## Figures and Tables

**Table 1:** Study inclusion and exclusion criteria.

Participants Were Eligible If They:	Patients Were Not Eligible If They:
- were ≥60 years and in long-term care (>4 weeks) - had dementia in accordance with DSM–5 - had either sleep/circadian rhythm disturbances, BPSD as identified by NPI–NH, or severely reduced ADL function - provided written informed consent if the participant had capacity or, if not, a written proxy informed consent from a legally authorized representative	- were blind or might otherwise not benefit from light - took part in another trial - had a condition contra-indicated to the intervention - had an advanced, severe medical disease/disorder and/or expected survival of less than 6 months, or other aspects that could interfere with participation - were psychotic or had a severe mental disorder

ADL = Activities of Daily Living, BPSD = Behavioral and Psychological Symptoms of Dementia; DSM-5 = Diagnostic and Statistical Manual of Mental Disorders–5; NPI–NH = Neuropsychiatric Inventory–Nursing Home Version.

**Table 2:** Baseline descriptive statistics.

	Control (N = 31)	Intervention (N = 30)	Total (N = 61)
Female	20 (64.5%)	23 (76.7%)	43 (70.5%)
Age—*M* (*Q*1, *Q3*)	82.0 (78.5, 87.5)	86.0 (83.0, 88.8)	84.0 (79.0, 88.0)
FAST			
*4*	0 (0.0%)	2 (6.7%)	2 (3.3%)
*5*	1 (3.3%)	1 (3.3%)	2 (3.3%)
*6*	21 (70.0%)	24 (80.0%)	45 (75.0%)
*7*	8 (26.7%)	3 (10.0%)	11 (18.3%)
CCI—*M* (*Q*1, *Q*3)	1.0 (1.0, 2.0)	2.0 (1.0, 2.0)	1.0 (1.0, 2.0)
MMSE—*M* (*Q*1, *Q*3)	4.0 (2.0, 9.0)	4.0 (1.0, 11.0)	4.0 (1.0, 10.0)
Psychotropic med. *—*M* (*Q*1, *Q*3)	3.0 (2.0, 3.0)	3.0 (2.0, 4.0)	3.0 (2.0, 4.0)
No. using hypnotics/sedatives ^†^	10 (32.3%)	12 (40.0%)	22 (36.1%)
No. with eye disease	6 (19.4%)	4 (13.3%)	10 (16.4%)
Dementia diagnoses			
Alzheimer’s	17 (55%)	16 (53%)	33 (54%)
Vascular	1 (3%)	2 (7%)	3 (5%)
Lewy body	1 (3%)	0 (0%)	1 (2%)
Other	1 (3%)	1 (3%)	2 (3%)
Unknown	9 (29%)	10 (33%)	19 (31%)

* Average number of ATC N-code drugs. ^†^ N05C drug in ATC system. M = median, Q1 = 25th percentile, Q3 = 75th percentile, FAST = Functional Assessment Staging Test, CCI = Charlson Comorbidity Index, MMSE = Mini-Mental State Exam.

**Table 3:** Spearman correlation coefficients between non-parametric and parametric RAR indicators at baseline.

	IS	IV	RA	M10	L5
Amplitude	0.66 ****	−0.52 ****	0.6 ****	0.46 ****	−0.29 *
MESOR	0.11	−0.21	−0.19	0.67 ****	0.54 *
Alpha	0.06 *	−0.26	0.15 *	−0.02	−0.11 ****
Beta	−0.18 *	0.26 ****	−0.10	0.00 *	0.02
F-statistic	0.81 ****	−0.58 ****	0.82 ****	0.48 ****	−0.49 ****
Acrophase	0.14 *	−0.23 *	−0.03	0.09 ****	0.09
Nadir	−0.41 ****	0.32 ****	−0.62 ****	0.18	0.66 ****

**** *p* < 0.0001, * *p* < 0.05. IS = inter-daily stability, IV = intra-daily variability, RA = relative amplitude, M10 = activity during the 10 most active hours, L5 = activity during the 5 least active hours. Estimated treatment effect.

**Table 4:** Results of mixed models, showing the week-by-group interactions.

	Week 8 × Intervention B (95 % CI)	Week 16 × Intervention B (95 % CI)	Week 24 × Intervention B (95 % CI)	N	N (id)	R^2^ (Fixed)	R^2^ (Total)	ICC
Non-Parametric Indicators								
IS	−0.01(−0.12–0.10)	−0.02(−0.15–0.10)	0.02(−0.10–0.13)	187	64	0.04	0.57	0.55
IV	−0.07(−0.22–0.09)	−0.02(−0.19–0.15)	−0.08(−0.23–0.07)	187	64	0.01	0.77	0.77
RA	−0.07(−0.19–0.05)	−0.05(−0.18–0.08)	−0.04(−0.16–0.09)	187	64	0.04	0.61	0.59
M10	11.3(−20.62–43.22)	−9.34(−44.51–25.83)	11.68(−20.04–43.40)	187	64	0.13	0.75	0.71
L5	8.47(−3.70–20.64)	−1.26(−14.65–12.13)	2.85(−9.25–14.94)	187	64	0.03	0.67	0.66
Extended Cosine Model								
Amplitude (ln)	−0.82(−1.81–0.17)	−0.41(−1.52–0.70)	−0.26(−1.28–0.75)	166	61	0.02	0.32	0.31
MESOR (ln)	−0.01(−0.44–0.43)	−0.21(−0.70–0.28)	−0.19(−0.64–0.25)	166	61	0.05	0.45	0.42
Acrophase *	−0.51(−1.39–0.37)	−1.02(−2.00–−0.05) ^†^	−0.59(−1.49–0.30)	163	61	0.10	0.63	0.59
Nadir *	0.41(−0.02–0.84)	0.01(−0.47–0.50)	−0.06(−0.50–0.38)	166	60	0.04	0.52	0.49
Alpha	−0.12(−0.38–0.14)	−0.14(−0.44–0.16)	−0.19(−0.46–0.08)	166	61	0.05	0.51	0.48
Beta (ln)	0.29(−0.65–1.24)	0.48(−0.57–1.53)	0.57(−0.40–1.54)	165	61	0.03	0.08	0.05
F-statistic (ln)	−0.4(−0.82–0.03)	−0.01(−0.49–0.47)	−0.06(−0.50–0.38)	166	61	0.05	0.78	0.77

Controlling for score on the Functional Assessment Staging Test (FAST). B = regression coefficient, N = number of observations, N(id) = number of participants, R2 (fixed) = marginal R2 (i.e., the proportion of variance explained by the fixed effects alone), R2 (total) = conditional R2 (i.e., the proportion of variance explained by fixed and random factors), ICC = intraclass correlation coefficient, IS = inter-daily stability, IV = intra-daily variability, RA = relative amplitude, M10 = activity during the 10 most active hours, L5 = activity during the 5 least active hours, ln = natural logarithm. * In decimal hours. ^†^ *p* = 0.04 without correction for false discovery rate.

## Data Availability

The data presented in this study are available from the corresponding author on reasonable request. The data are not publicly available due to the risk of compromising the privacy of participating individuals.

## Supplementary Materials

The following are available online at https://www.mdpi.com/article/10.3390/clockssleep3030032/s1, Table S1: “Light measurements in 8 dementia units after installation of light fixtures”; Table S2: “Spectral power distributions for participating dementia units”; and Table S3: “Rest activity rhythm measures by week of study for each treatment group”.
