# Supplementary material for: The Effect of Bright Light Treatment on Rest–Activity Rhythms in People with Dementia: A 24-Week Cluster Randomized Controlled Trial
Source: Clocks Sleep. 2021 Sep 13;3(3):449–64. doi: 10.3390/clockssleep3030032 (PMC8482074; doi:10.3390/clockssleep3030032)
Supplement: Supplementary file 1 [file clockssleep-03-00032-s001.zip › Table S1.pdf]

Table S1. Light measurements in 8 dementia units after installation of light fixtures.

	Control (4 units)	Intervention (4 units)
Photopic illuminance (lx)		
Mean (SD)	242 (101)	1039 (225)
Range	134 - 368	722 - 1242
Irradiance		
Mean (SD)	860 (469)	3472 (793)
Range	410 - 1496	2359 - 4172
S-cone-opic irradiance		
Mean (SD)	82 (56)	694 (143)
Range	33 - 163	495 - 801
M-cone-opic irradiance		
Mean (SD)	290 (138)	1428 (297)
Range	151 - 470	1008 - 1684
L-cone-opic irradiance		
Mean (SD)	397 (165)	1680 (364)
Range	221 - 602	1167 - 2008
Rhodopic irradiance		
Mean (SD)	207 (123)	1259 (247)
Range	93 - 375	914 - 1454
Melanopic irradiance		
Mean (SD)	164 (108)	1100 (209)
Range	67 - 313	812 - 1261
Melanopic EDI		
Mean (SD)	124 (82)	779 (142)
Range	50 - 236	612 - 951

Measured equidistant to all walls, vertically at 1.2 m height, averages of four directions. Irradiance measured in mWm^2. SD = standard deviation. EDI = Equivalent daylight (D65) illuminance.
