# Supplementary material for: The Effect of Bright Light Treatment on Rest–Activity Rhythms in People with Dementia: A 24-Week Cluster Randomized Controlled Trial
Source: Clocks Sleep. 2021 Sep 13;3(3):449–64. doi: 10.3390/clockssleep3030032 (PMC8482074; doi:10.3390/clockssleep3030032)
Supplement: Supplementary file 1 [file clockssleep-03-00032-s001.zip › Table S3.pdf]

Table S3. Rest activity rhythm measures by week of study for each treatment group.

RAR-measure Median (Q1, Q3)	Baseline Control (N=31)	Baseline Intervention (N=30)	Week 8 Control (N=27)	Week 8 Intervention (N=20)	Week 16 Control (N=22)	Week 16 Intervention (N=18)	Week 24 Control (N=21)	Week 24 Intervention (N=25)
IS	0.50 (0.21, 0.67)	0.49 (0.42, 0.57)	0.41 (0.28, 0.60)	0.49 (0.36, 0.57)	0.43 (0.20, 0.56)	0.45 (0.31, 0.56)	0.44 (0.27, 0.56)	0.55 (0.44, 0.61)
IV	1.03 (0.68, 1.40)	1.07 (0.86, 1.30)	1.04 (0.71, 1.26)	0.92 (0.64, 1.12)	1.04 (0.78, 1.37)	0.97 (0.68, 1.14)	0.99 (0.79, 1.31)	0.97 (0.81, 1.25)
RA	0.63 (0.43, 0.83)	0.78 (0.62, 0.86)	0.71 (0.42, 0.82)	0.80 (0.62, 0.84)	0.60 (0.39, 0.73)	0.77 (0.59, 0.84)	0.61 (0.49, 0.79)	0.81 (0.57, 0.86)
M10	111.07 (68.20, 158.55)	149.54 (117.91, 212.87)	117.65 (81.90, 142.32)	130.95 (88.83, 257.34)	112.19 (68.07, 137.82)	108.45 (80.35, 192.83)	122.48 (86.48, 142.39)	159.39 (100.79, 216.78)
L5	20.75 (8.33, 30.67)	21.48 (7.56, 35.44)	21.96 (11.35, 38.36)	14.56 (9.88, 38.92)	23.24 (14.44, 39.96)	16.62 (9.90, 26.19)	22.43 (10.13, 35.90)	20.07 (8.35, 44.55)
Amplitude	11.95 (3.65, 34.95)	16.61 (9.09, 40.28)	34.09 (9.03, 57.79)	19.28 (9.45, 30.74)	23.08 (10.47, 51.53)	16.89 (7.57, 86.61)	15.50 (7.78, 61.15)	17.27 (9.99, 63.24)
Mesor	9.91 (7.22, 16.30)	12.07 (7.16, 19.01)	11.40 (8.83, 12.95)	12.35 (9.42, 19.26)	14.46 (10.29, 19.36)	11.45 (7.49, 13.30)	12.81 (10.13, 19.15)	10.62 (7.34, 14.35)
Acrophase	15.65 (14.54, 16.10)	14.71 (14.39, 15.85)	15.77 (14.70, 16.54)	14.79 (14.22, 16.00)	16.02 (15.26, 17.12)	14.55 (14.32, 15.22)	15.92 (14.78, 16.76)	14.50 (14.09, 15.73)
Nadir	1.03 (0.58, 1.51)	0.72 (0.40, 1.18)	0.85 (0.30, 1.26)	0.95 (0.72, 1.43)	1.36 (0.26, 1.84)	0.70 (0.33, 1.29)	0.89 (0.54, 1.41)	0.78 (0.45, 1.19)
Alpha	-0.25 (-0.37, 0.02)	-0.11 (-0.36, 0.03)	-0.15 (-0.28, 0.59)	-0.03 (-0.17, 0.09)	-0.05 (-0.27, 0.47)	-0.04 (-0.28, 0.10)	-0.15 (-0.25, 0.32)	-0.10 (-0.34, 0.02)
Beta	9.11 (4.99, 21.76)	11.01 (4.83, 18.64)	8.12 (4.05, 12.39)	7.76 (5.46, 11.31)	5.65 (3.57, 14.26)	11.28 (5.87, 21.75)	6.78 (3.16, 18.11)	18.71 (4.52, 32.86)
Pseudo-F statistic	675.10 (174.54, 1412.73)	912.12 (575.76, 1467.22)	1026.24 (307.26, 1849.15)	894.79 (433.29, 2209.86)	934.32 (353.45, 1359.26)	1115.45 (706.61, 2177.94)	761.31 (339.69, 1285.68)	1121.09 (837.12, 1865.15)

IS = interdaily stability, IV = intradaily variability, RA = relative amplitude, M10 = activity during the 10 most active hours, L5 = activity during the 5 least active hours, Q1 =25. percentile, Q3 = 75. percentile. Includes outliers removed during the analysis stage.
